# Social communication deficits: Specific associations with Social Anxiety Disorder

**DOI:** 10.1016/j.jad.2014.09.040

**Published:** 2015-02-01

**Authors:** Georgia Halls, Peter J. Cooper, Cathy Creswell

**Affiliations:** a School of Psychology and Clinical Language Sciences University of Reading, UK; b Department of Psychology, University of Stellenbosch, Cape Town, South Africa

**Keywords:** Social Anxiety Disorder, Child, Social communication, Autism spectrum, Anxiety

## Abstract

**Background:**

Social communication deficits are prevalent amongst children with anxiety disorders; however whether they are over-represented specifically among children with Social Anxiety Disorder has not been examined. This study set out to examine social communication deficits among children with Social Anxiety Disorder in comparison to children with other forms of anxiety disorder.

**Methods:**

Parents of 404 children with a diagnosed anxiety disorder completed the Social Communication Questionnaire (SCQ; Rutter, M., Bailey, A., Lord, C., 2003. The Social Communication Questionnaire – Manual. Western Psychological Services, Los Angeles, CA). Children with a diagnosis of Social Anxiety Disorder (*n*=262) and anxious children without Social Anxiety Disorder (*n*=142) were compared on SCQ total and subscale scores and the frequency of participants scoring above clinical cut-offs.

**Results:**

Children with Social Anxiety Disorder scored significantly higher than anxious children without Social Anxiety Disorder on the SCQ total (*t*(352)=4.85, *p*<.001, *d*=.55, *r*=.27), Reciprocal Social Interaction (*t*(351)=4.73, *p*<.001, *d*=.55, *r*=.27), communication (*t*(344)=3.62, *p*<.001, *d*=.43, *r*=.21) and repetitive, restrictive and stereotyped behaviors subscales (*t*(353)=3.15, *p*=.002, *d*=.37, *r*=.18). Furthermore, children with Social Anxiety Disorder were three times more likely to score above clinical cut-offs.

**Limitations:**

The participants were a relatively affluent group of predominantly non-minority status. The social communication difficulties measure relied on parental report which could be influenced by extraneous factors.

**Conclusions:**

Treatments for Social Anxiety Disorder may benefit from a specific focus on developing social communication skills. Future research using objective assessments of underlying social communication skills is required.

## 1 Introduction

Anxiety disorders during childhood are common, serious, and are associated with poor life course consequences (e.g. Costello et al., 2004; Essau et al., 2000; Ezpeleta et al., 2001) and substantial health and social costs (Bodden et al., 2008). Social Anxiety Disorder, in particular, has a high prevalence (Costello et al., 2004) and is linked with serious long term consequences, including depression (Beesdo et al., 2007). Recent studies have demonstrated that children with Social Anxiety Disorder have poorer outcomes than children with other forms of anxiety from generic or transdiagnostic anxiety treatments (Compton et al., 2014; Ginsburg et al., 2011; Kerns et al., 2013). However, treatments which specifically target Social Anxiety Disorder appear to be associated with somewhat better outcomes (Doehrmann et al., 2013; Mayo-Wilson et al., in preparation). These treatments are often more intensive, yet the content is typically broadly similar to the generic treatments; a key difference being increased opportunities for rehearsal of social skills (Beidel et al., 2000; Spence et al., 2000).

Early social communication difficulties have been implicated in the development of Social Anxiety Disorder. Specifically, it has been suggested that a lack of social skills may lead to negative reactions from others, which promote negative beliefs and avoidant behaviors in social situations (Rapee and Spence, 2004). Consistent with this hypothesis, children with autism spectrum disorder have elevated social anxiety symptoms compared to healthy peers (Kuusikko et al., 2008), which is accounted for by the degree of their social communication difficulties (Bellini, 2004). Also consistent with this hypothesis, compared to non-anxious children, children with Social Anxiety Disorder have been found to have lower self and peer ratings of social competence, as well as poorer social skills on behavioral assessments in the laboratory and in school (Beidel et al., 1999; Spence et al., 1999). However, evaluating social skills in situations where children are anxious introduces a confound, which makes it difficult to ascertain whether group differences reflect social communication skill deficits or a lack of social confidence. For example, Cartwright‐Hatton et al. (2003) reported that children with higher levels of social anxiety only scored higher than others on observer ratings of how nervous they appeared, and not on other aspects of social skill, although the children gave more negative appraisals of their performance.

One approach to teasing apart social skills deficits and social anxiety is to consider the social communication difficulties that may underlie restricted social behaviors. Social communication deficits are a core trait of Autistic Spectrum Disorder, and include difficulties with communication of cognitive and emotional information through facial expression, gesture, and prosody and through implicit understanding of pragmatics and theory of mind (e.g. Tanguay et al., 1998). Of particular note is one study with a community population, in which children with high levels of social anxiety were rated by teachers as being less skilled in social tasks that involved insight into others׳ mental states, compared to their low-anxious peers (Banerjee and Henderson, 2001). Few studies have examined underlying social communication difficulties among children with Social Anxiety Disorder specifically, but a higher frequency of social communication difficulties has been found among groups of children with a range of anxiety disorders, compared to their non-anxious peers (van Steensel et al., 2013).

This study examines the degree to which social communication difficulties are specifically associated with Social Anxiety Disorder. In order to address the question of specificity, we compared social communication deficits among children meeting diagnostic criteria for Social Anxiety Disorder and children with other anxiety disorders. As anxiety disorders in children are commonly comorbid (e.g. Kendall et al., 2010), we accounted for this in our analyses, as well as other potential confounds, such as gender, age, ethnicity and any potential overlap in the assessment of social anxiety and social communication difficulties.

In summary, this study investigated the hypothesis that children with Social Anxiety Disorder will have significantly more social communication deficits, or ASD traits, than children with other forms of anxiety disorder.

## 2 Methods

### 2.1 Participants

Four-hundred and four clinically anxious children, aged 6–13 years, were assessed before commencing treatment. 64.9% (*n*=262) of the children met diagnostic criteria for current Social Anxiety Disorder (SOCANX), and 35.1% (*n*=142) met criteria for another anxiety disorder but not Social Anxiety Disorder (NONSOCANX). The two groups were well balanced on child age, gender, ethnicity and socio-economic status (see Table 1).

All participating children were recruited through referrals by local health and education service personnel to the Berkshire Child Anxiety Clinic at the University of Reading. Children were assessed by graduate psychologists using the Anxiety Disorders Interview Schedule for DSM-IV: Child and Parent version (ADIS-C/P (see below)) and were included on the basis of having an anxiety disorder as their principal diagnosis. The clinical service does not offer treatment to children with diagnosed autistic spectrum disorders (as alternative services are available), so referrals were accepted on the basis that an autistic spectrum disorder had not been identified.

### 2.2 Procedure

Ethical approval was granted from the University of Reading and Berkshire (NHS) Research Ethics Commitees. All potential participants received information in writing and from discussions with the research team, and provided written consent. Participating children and their primary caregiver completed initial diagnostic interviews and symptom questionnaires either in University clinic rooms or in satellite clinics in their locality.

### 2.3 Measures

#### 2.3.1 Diagnoses

Children were assigned diagnoses on the basis of the Anxiety Disorders Interview Schedule for DSM IV for Children-Child and Parent Versions (ADIS-C/P; Silverman and Albano, 1996), a structured diagnostic interview with well-established psychometric properties (Silverman et al., 2001). When children met symptom criteria for a diagnosis (based on either child or parent report) they were assigned a clinical severity rating (CSR) from 0 (complete absence of psychopathology) to 8 (severe psychopathology). The higher CSR of the two was taken. As is conventional, only those who met symptom criteria with a CSR of 4 or more (moderate psychopathology) were considered to meet diagnostic criteria.

Assessors (psychology graduates) were trained on the administration and scoring of the ADIS-C/P through verbal instruction, listening to assessment audio-recordings and participating in diagnostic consensus discussions. The first 20 interviews conducted were then discussed with a consensus team, led by an experienced diagnostician (Consultant Clinical Psychologist). The assessor and the consensus team independently allocated diagnoses and CSRs. Following the administration of 20 child or 20 parent interviews, inter-rater reliability for each assessor was checked, and if assessors achieved reliability of at least .85 they were then required to discuss just one in six interviews with the consensus team (these ongoing checks were conducted to prevent inter-rater drift). Overall reliability for the team was excellent. Reliability for ADIS-C/P diagnosis was .98 (child report), .98 (mother report); and for CSRs .99 (child report), .99 (mother report).

#### 2.3.2 Symptoms measures

The Spence Children׳s Anxiety Scale (SCAS-c/p; Nauta et al., 2003; Spence, 1998) was used to assess child and parent reported child anxiety symptoms. The child version requires children to rate how often they experience each of 38 anxiety symptoms, presented alongside six positive filler items on a 4-point scale from 0 (*never*) to 3 (*always*). Both versions have demonstrated good concurrent validity and internal consistency (Nauta et al., 2004; Spence, 1998). Internal consistency based on data from the current sample was good (Cronbach׳s alpha (*α*)=.92 for SCAS-C and .89 for SCAS-P).

The life-time version of the Social Communication Questionnaire (SCQ) is a 40 item parent-reported checklist of ‘yes or no’ questions from the Autism Diagnostic Interview-Revised (ADI-R). The SCQ has three subscales: Reciprocal Social Interaction (13 items), communication (8 items) and restricted and repetitive behaviors (RRBI; 6 items). The SCQ has demonstrated high reliability ranging from .84 to .93 (Rutter et al., 2003), with good internal consistency from the current sample (*α*=.86; subscale scores were lower for communication and RRBI likely due to the restricted variance from dichotomous responses to a low number of items; Social *α*=.85, communication *α*=.56, RRBI *α*=.42). Total scores of 15 or more indicate a likelihood of ASD (Rutter et al., 2003). The SCQ has been found to have high sensitivity and specificity when differentiating autism from other ASDs and no spectrum diagnoses (.90 and .86 respectively; Chandler et al., 2007) and performs better in this regard than other questionnaire measures of social communication (Charman et al., 2007).

## 3 Results

### 3.1 Preliminary analyses

Continuous data were screened in relation to the assumptions of parametric tests (Tabachnick and Fidell, 2013). The distribution of the SCQ total was highly positively skewed (*z*=1.20), as were the subscales, Reciprocal Social Interaction (*z*=2.24), communication (z =1.30) and RRBI (*z*=1.26). The majority of scales fitted a normal distribution when square-root transformations were conducted, with the exception of the SCQ Reciprocal Social Interaction Subscale which remained skewed. In this case both parametric and non-parametric tests were performed to ensure findings were robust. As the results were consistent, the parametric test results are reported for simplicity.

Preliminary analyses were conducted to identify potential confounding variables. There was no significant association between anxiety disorder group or age in months (*t*(402)=.36, *p*=.49), socioeconomic status (*χ*^2^ (2, *N=*356)=5.04, *p*=.08), ethnicity (*χ*^2^ (1, *N=*393)=2.17, *p*=.14), or gender (*χ*^2^ (1, *N=*404)=1.95, *p*=.16). However, children with Social Anxiety Disorder were more likely to have comorbid Mood Disorders (*χ*^2^ (1, *N=*404)=15.23, *p*<.001), Separation Anxiety Disorder (*χ*^2^ (1, *N=*404)=10.36, *p*<.001), and Generalized Anxiety Disorder (*χ*^2^ (1, *N=*404)=32.96, *p*<.001) (see Table 2), than the non-socially anxious group. Analyses were therefore rerun excluding children with each of these comorbid diagnoses to establish whether associations with Social Anxiety Disorder remained. This approach was taken, rather than entering diagnostic or self-report scores as covariates due to concerns about colinearity. Significantly more children in the No Social Anxiety Disorder group also had a diagnosis of Panic Disorder without Agoraphobia (*χ*^2^ (1, *N=*404)=5.46, *p*=.019) and Anxiety Disorder Not Otherwise Specified (*χ*^2^ (1, *N=*404)=8.37, *p*=.008); however the frequencies were low in both groups (*n*= 10 and *n*=14) so no further analyses were conducted.

### 3.2 Hypothesis testing

As shown in Table 1, children with Social Anxiety Disorder scored significantly higher than the No Social Anxiety group on the SCQ total score (*t*(352)=4.85, *p*<.001, *d*=.55, *r*=.27), Reciprocal Social Interaction (*t*(351)=4.73, *p*<.001, *d*=.55, *r*=.27), communication (*t*(344)=3.62, *p*<.001, *d*=.43, *r*=.21) and RRBI subscale scores (*t*(353) =3.15, *p*=.002, *d*=.37, *r*=.18). Furthermore, children with Social Anxiety Disorder were significantly more likely to score above the cut-off threshold for autism spectrum disorder on the SCQ (*χ*^2^ (1, *N=*354)=5.87, *p*=.015), with three times as many children in the Social Anxiety Disorder group than the No Social Anxiety Group scoring above clinical cut-offs.

The analyses were repeated including only those children who had Social Anxiety Disorder as a primary diagnosis (versus those without a diagnosis of Social Anxiety Disorder). The pattern of results was consistent with all those reported above. Sensitivity analyses were also conducted excluding children with (i) Separation Anxiety Disorder (*n*=234), (ii) Generalized Anxiety Disorder (*n*=260), and (iii) Mood Disorder (*n*=57). Group differences in the total SCQ score and the Reciprocal Social Interaction subscale were robust to this reduction in the available sample, although differences in SCQ communication and RRBI were no longer significant across all of these follow-up analyses (see Table 3). Notably, when children with comorbid GAD were excluded there were no significant differences in anxiety symptom severity between children with and without Social Anxiety Disorder (SCAS-C, *t*(133)=1.62, *p*=.11, *d*=.28, *r*=.14; SCAS-P, *t*(132)=1.74, *p*=.08, *d*=.30, *r*=.15), suggesting that differences in symptom severity do not account for the significant group differences in SCQ scores.

As three items in the SCQ could feasibly refer to symptoms of social anxiety, a final set of sensitivity analyses were run after removing these three items from the SCQ to ensure that group differences were not accounted for by potentially overlapping questions. These questions examined use of socially inappropriate questions, having a best friend and being able to engage in a friendly talk (without the intention to gain anything). Again, the pattern of results was consistent with those reported above.

## 4 Discussion

Consistent with predictions, compared to children with a non-social form of anxiety disorder, children with Social Anxiety Disorder were rated as having a higher level of social communication deficits across all the domains assessed, i.e. social interaction difficulties, communication difficulties, and restricted and repetitive behaviors. These group differences did not seem to be accounted for by overlapping symptoms (such as asking socially inappropriate questions and holding a friendly conversation), as when items reflecting these symptoms were removed from the social communication deficit measure, the group effects were maintained. Furthermore, group differences on the total SCQ score were also maintained when children with common comorbid conditions were excluded from the analyses and when overall anxiety symptom severity was balanced across groups. Despite there being alternative services for children with autistic spectrum disorders and the participating service not delivering services to these children, 26 children in the current sample scored above the clinical cut-offs for social communication deficits, indicating probable ASD. Three times as many children in the Social Anxiety Disorder group scored above the SCQ clinical cut-offs than for those with other anxiety disorders but not Social Anxiety Disorder.

The findings are consistent with the suggestion that, for a significant subgroup of children with Social Anxiety Disorder, social communication deficits may underlie the anxiety disorder (Rapee and Spence, 2004). However, prospective longitudinal studies are needed to establish the direction of the association between social communication deficits and social anxiety. Recent findings that children with Social Anxiety Disorder show low rates of recovery from generic cognitive behavior therapy have led to the suggestion that this may have been a result of a lack of within session exposure (e.g. Compton et al., 2014). However, along with recent findings that social communication deficits predict poor outcomes from cognitive behavioral therapy for children with anxiety disorders (Puleo and Kendall, 2011), the results of the current study indicate that treatments for Social Anxiety Disorders may, in some cases, require a specific focus on developing children׳s social communication abilities.

Strengths of this study include the large clinical population which allowed consideration of specific anxiety disorders, diagnosed on the basis of systematic and reliable assessments. However, the findings should also be interpreted with various limitations in mind. The participants were predominantly from a reasonably affluent social group of non-minority status, limiting the generalizability of the findings. Although a widely used and evaluated measure of social communication difficulties was used, this measure relied on parental report which could be influenced by extraneous factors (such as parental anxiety). Furthermore, future studies would benefit from using objective tests of social communication deficits to verify the current findings and to potentially help identify specific underlying processes that may be pertinent to address in the treatment of Social Anxiety Disorder.

## 5 Conclusion

Children with a diagnosis of Social Anxiety Disorder had higher levels of social communication deficits compared to children with non-social forms of anxiety disorder. The findings suggest that treatments for Social Anxiety Disorder may benefit from the inclusion of a specific focus on social communication difficulties.

## Role of funding source

Dr. Creswell was funded by MRC Clinician Scientist Fellowship (G0601874). Participants were assessed within a treatment trial funded by the MRC–NIHR partnership (09/800/17).

## Conflict of interest

None.

## Figures and Tables

**Table 1:** Descriptive characteristics.

	Social Anxiety Disorder (*N*=262)		No Social Anxiety Disorder (*N*=142)	
	% (*n*)			

Gender (male)	46.95 (123)	54.22 (77)	*χ*^2^ (1)=1.95	
Ethnicity (White British)	85.50 (224)	83.10 (118)	*χ*^2^ (1)=2.17	
Socio-economic status (higher/professional)	24.80 (65)	26.06 (37)	*χ*^2^ (1)=5.04	
SCQ (above clinical cut off)	878 (23)	2.11 (3)	*χ*^2^ (1)=5.87⁎	
	Mean (SD)			

Age (months)	121.57 (19.41)	120.85 (18.97)	*t*(367)=.363	
SCAS-p	43.40 (16.69)	34.00 (14.34)	*t*(367)=5.19⁎⁎⁎	
SCAS-c	41.78 (19.31)	35.76 (16.54)	*t*(383)=3.55⁎⁎⁎	
SCQ	6.91 (5.28)	4.26 (4.35)	*t*(351)=4.26⁎⁎⁎	
SCQ RRBI	.87 (.95)	.54 (.88)	*t*(353)=2.84⁎⁎	
SCQ RSID	1.99 (2.56)	.95 (2.26)	*t*(367)=3.97⁎⁎⁎	
SCQ C	1.56 (1.67)	.97 (1.35)	*t*(344)=3.34⁎⁎	

*Note*: SD=standard deviation. SCAS-p=Spence Children׳s. Anxiety Scale-parent report. SCAS-c=Spence Children׳s Anxiety Scale- child report. SCQ=Social Communication Questionnaire. SCQ RRBI=Social Communication Questionnaire Restricted, Repetitive and Stereotyped patterns of Behavior. SCQ RSID=Social Communication Questionnaire Reciprocal Social Interaction Difficulties Subscale. SCQ C=Social Communication Questionnaire Communication Difficulties Subscale.

⁎ *p*<.05.

⁎⁎ *p*<.01.

⁎⁎⁎ *p*<.001.

**Table 2:** Frequency of comorbid anxiety disorders.

	Social Anxiety Disorder *N*= 262	No Social Anxiety Disorder *N*= 142	Chi-square
	% (*n*)		
Separation Anxiety Disorder	63.7 (167)	47.1 (67)	*χ*^2^ (1)=10.36⁎⁎
Specific Phobia Animal Type	43.9 (115)	45.8 (65)	*χ*^2^ (1)=.13
Specific Phobia Natural Environment Type	18.7 (49)	.07 (18)	*χ*^2^ (1)=2.42
PD w/o Agoraphobia	.01 (3)	.05 (7)	*χ*^2^ (1)=5.46⁎
PD with Agoraphobia	.01 (2)	.01 (1)	*χ*^2^ (1)=.000
Agoraphobia w/o PD	.05 (12)	.08 (12)	*χ*^2^ (1)=2.47
GAD	74.4 (195)	45.7 (65)	*χ*^2^ (1)=32.96⁎⁎⁎
Obsessive Compulsive Disorder	.04 (11)	.03 (4)	*χ*^2^ (1)=.49
Selective Mutism	.01 (2)	.00 (0)	*χ*^2^ (1)=1.09
Anxiety Disorder NOS	.02 (4)	.07 (10)	*χ*^2^ (1)=8.37⁎⁎

*Note:* PD=Panic Disorder. w/o=without. GAD=Generalized Anxiety Disorder. NOS=not otherwise specified.

⁎ *p*<.05.

⁎⁎ *p*<.01.

⁎⁎⁎ *p*<.001.

**Table 3:** Statistical comparisons of children with Social Anxiety Disorder vs. those without, after excluding participants with common comorbid disorders.

	Excluded disorder		
	GAD	Separation Anxiety	Mood Disorder
SCQ	*t*(124)=1.995⁎	*t*(144)=2.866⁎⁎	*t*(299)=3.771⁎⁎⁎
(effect size; *r*)	(.21)	(.21)	(.22)
SCQ RSID	*t*(124)=2.987⁎⁎	*t*(143)=2.609⁎	*t*(298)=3.847⁎⁎⁎
(*r*)	(.21)	(.13)	(.18)
SCQ C	*t*(122)=1.685	*t*(140)=1.896	*t*(293)=2.748⁎
(*r*)	(.11)	(.15)	(.15)
SCQ RRBI	*t*(124)=.011	*t*(145)=2.333⁎	*t*(300)=2.384⁎
(*r*)	(.01)	(.20)	(.13)

SCQ (above clinical cut off)			
% (*n*)	10.00 (6)	10.34 (9)	6.28 (12)
Comparison of proportion with SCQ scores above vs. below clinical cut-off	*χ*^2^ (1)=2.57	*χ*^2^ (1)=1.29	*χ*^2^ (1)=1.86

*Note:* SCQ=Social Communication Questionnaire. SCQ RSID=Social Communication Questionnaire Reciprocal Social Interaction Difficulties Subscale. SCQ C=Social Communication Questionnaire Communication Difficulties Subscale. SCQ RRBI=Social Communication Questionnaire Restricted, Repetitive and Stereotyped patterns of Behavior. GAD=Generalized Anxiety Disorder.

⁎ *p*<05.

⁎⁎ *p*<01.

⁎⁎⁎ *p*<001.
